# Predator becomes prey: Martial eagle predation of lion cubs in the greater Mara region, Kenya

**DOI:** 10.1002/ece3.70148

**Published:** 2024-09-13

**Authors:** Richard Stratton Hatfield, Lemein Parmuntoro, Simon Thomsett, Patrick Reynolds, Nicholas B. Elliot

**Affiliations:** ^1^ Wageningen University and Research Wageningen The Netherlands; ^2^ The Kenya Bird of Prey Trust Naivasha Kenya; ^3^ The Bird of Prey Trust Borssele The Netherlands; ^4^ Governor's Camps Narok Kenya; ^5^ Wildlife Counts, Langata Link Complex Nairobi Kenya

**Keywords:** interspecific killing, intraguild predation, *Panthera leo*, *Polemaetus bellicosus*, top predator

## Abstract

In many intact African savannah ecosystems, martial eagles are the top avian predator, while lions are the top terrestrial predator. Here, we report seven records of martial eagle predation or attempted predation of lion cubs in the greater Mara region, Kenya. These events resulted in the death of nine lion cubs, most of which were at least partially consumed, and are the first detailed records of this behaviour to be published. While these observations represent intraguild interactions, we suggest that they reflect neither intraguild predation nor interspecific killing. Rather, the ecology of both species coupled with the details of our records suggest that martial eagles opportunistically kill lion cubs purely to eat them. We hope that by publishing these records we will encourage others to share their observations of raptors predating on large mammalian carnivores, thus improving our understanding of a behaviour that we suspect may be more widespread than the current lack of evidence suggests.

## Graphical abstract

In many intact African savannah ecosystems, martial eagles are the top avian predator, while lions are the top terrestrial predator. Here, we report seven records of martial eagle predation or attempted predation of lion cubs in the greater Mara region, Kenya. While these observations represent intraguild interactions, we suggest that martial eagles opportunistically kill lion cubs purely to eat them and that this behaviour is more widespread than previously understood.

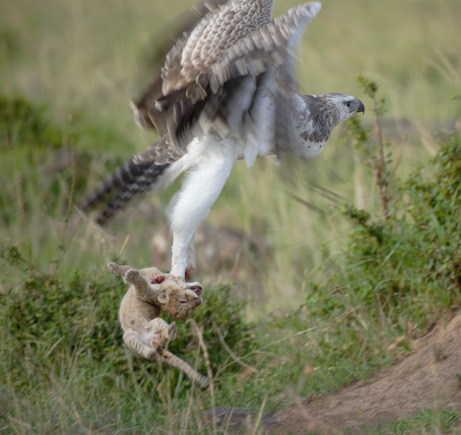

## 1 INTRODUCTION

Top predators are broadly defined as species that occupy the highest trophic niches (Sergio et al., 2014). They predate on other species and are rarely predated on in their adult form (Wallach et al., 2015). Intraguild interactions between top predators are often characterised by aggressive behaviour that results in mortality. Both species may kill each other (symmetrical), or one species may kill the other (asymmetrical) and in some cases only the young are killed (Palomares & Caro, 1999). These interactions have traditionally been defined as ‘interspecific killing’ (where potentially competing species are killed, but there is no energetic gain for the predator) or ‘intraguild predation’ (where potentially competing species are killed and eaten) (Lourenço et al., 2014; Polis et al., 1989). These lethal interactions generally occur among species that share a significant dietary overlap and thus are frequently considered a form of killing to pre‐emptively reduce intraguild competition (Palomares & Caro, 1999).

Interspecific killing has been documented in 97 pairwise interactions involving mammalian carnivores, but in many cases information on consumption is not provided (Lourenço et al., 2014; Palomares & Caro, 1999) and energy gained through feeding is frequently regarded as an incidental benefit rather than a primary motivation of the killing (Polis & Holt, 1992; Ritchie & Johnson, 2009). Interspecific killing and intraguild predation have received much attention among similar species groups (Sergio & Hiraldo, 2008), perhaps reflecting carnivorans tendency to interact more with species from the same taxonomic families (Donadio & Buskirk, 2006). Studies that have explored these interactions among taxonomically distinct top predator species groups are more uncommon, no doubt reflecting the paucity of observations describing and quantifying these interactions.

Here, we report seven records where a top avian predator, the martial eagle (*Polemaetus bellicosus*), killed or attempted to kill the young of a top mammalian predator, the African lion (*Panthera leo*). Although the two species are obligate carnivores that occupy the highest trophic positions within an ecosystem, they share little niche overlap and are not direct competitors. Martial eagles rarely scavenge, and their diet consists of a wide variety of medium‐ to large‐sized birds, reptiles and mammals ranging in weight from 200 to ~7500 g (Hatfield, 2018; Hatfield et al., 2024; Naude et al., 2019). Lions meanwhile will readily scavenge and generally feed on medium to large ungulates within a weight range of 190 to 550 kg (Hayward & Kerley, 2005).

## 2 THE MAASAI MARA

**FIGURE 1:**
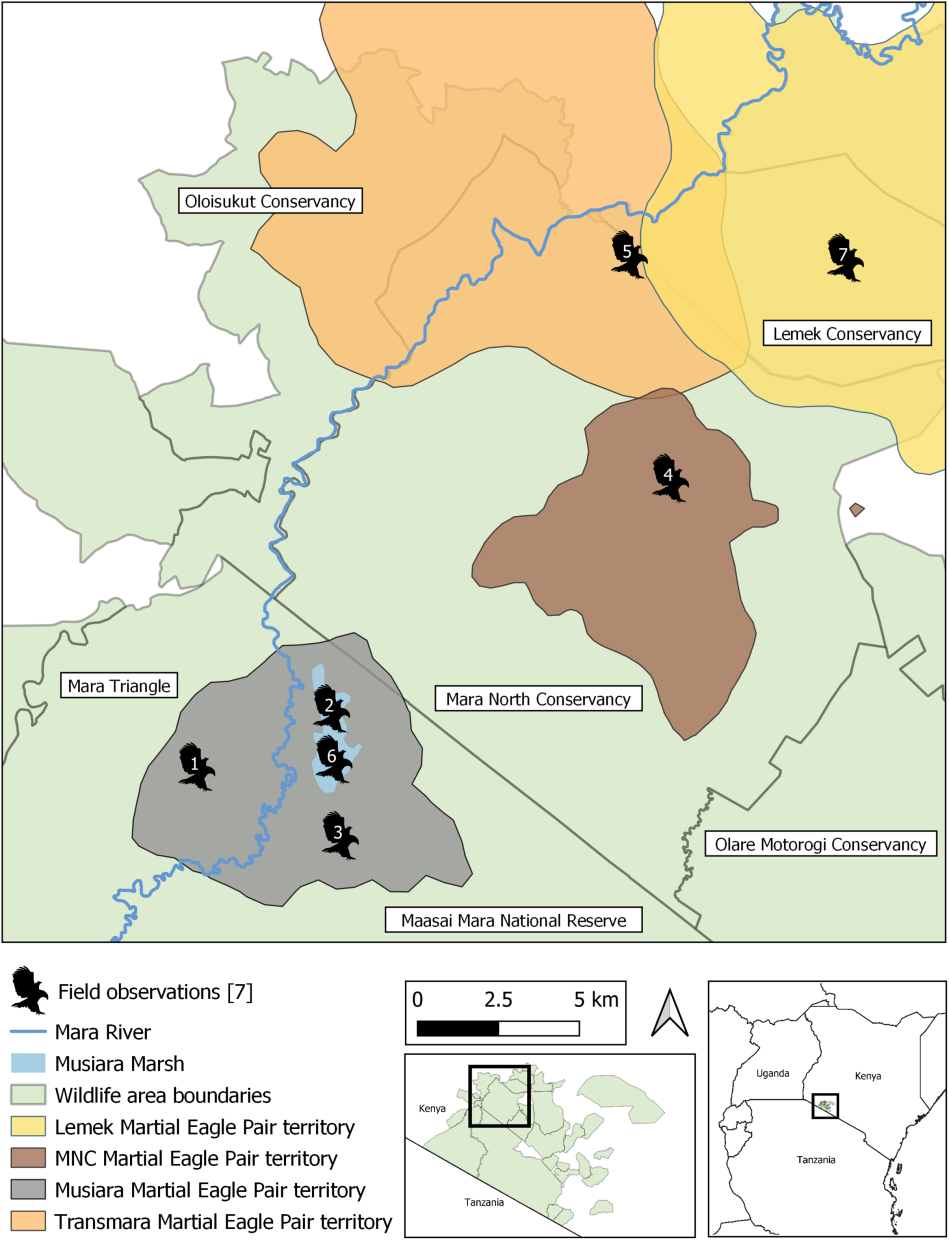
The locations of the seven field records of martial eagle predation or attempted predation of lion cubs in the greater Mara region, Kenya. Each record is plotted in relation to estimated martial eagle pair territory boundaries. These boundaries are kernel density estimated home ranges taken from work described in Hatfield (2018). Note how the seven records are distributed within the boundaries of four territorial pairs.

Our records all come from the greater Mara region in southwestern Kenya (N −1.3, E 35.0, Figure 1). The area is characterised by open grasslands interspersed with woodlands and shrublands, and surface water is available from numerous permanent and seasonal rivers (Oindo et al., 2003). It comprises roughly 2500 km^2^ of wildlife areas under either private or local government management. The ecosystem supports a high abundance of ungulates and therefore predators, with lions occurring at a density of 17 individuals >1 year old/100 km^2^ (Elliot & Gopalaswamy, 2017). This high lion density, coupled with large volumes of tourists and open habitats, provides unique opportunities to observe and record potentially rare behaviours. Martial eagle population densities are unknown within the greater Mara, but ongoing work estimates a minimum population of 20 adult breeding pairs, and 20 telemetered individuals have a mean territory size of approximately 174.5 km^2^ (range: 61.7–654.9 km^2^; Hatfield, 2018). The ecosystem supports an unknown number of nomadic juvenile and subadult martial eagles.

## 3 FIELD RECORDS

We provide seven records that detail martial eagle predation of nine lion cubs, and one attempted predation in Kenya's greater Mara region. All records were opportunistic observations collected by guides and/or tourists and are supported with documentation in the form of photographs, video and/or detailed notes from experienced observers, which also allowed us to provide approximate locations of each record (Figure 1). All seven records were distributed within the boundaries of four telemetered territorial martial eagle pairs, albeit records 1–3 were observed prior to the onset of the telemetry study.

**FIGURE 2:**
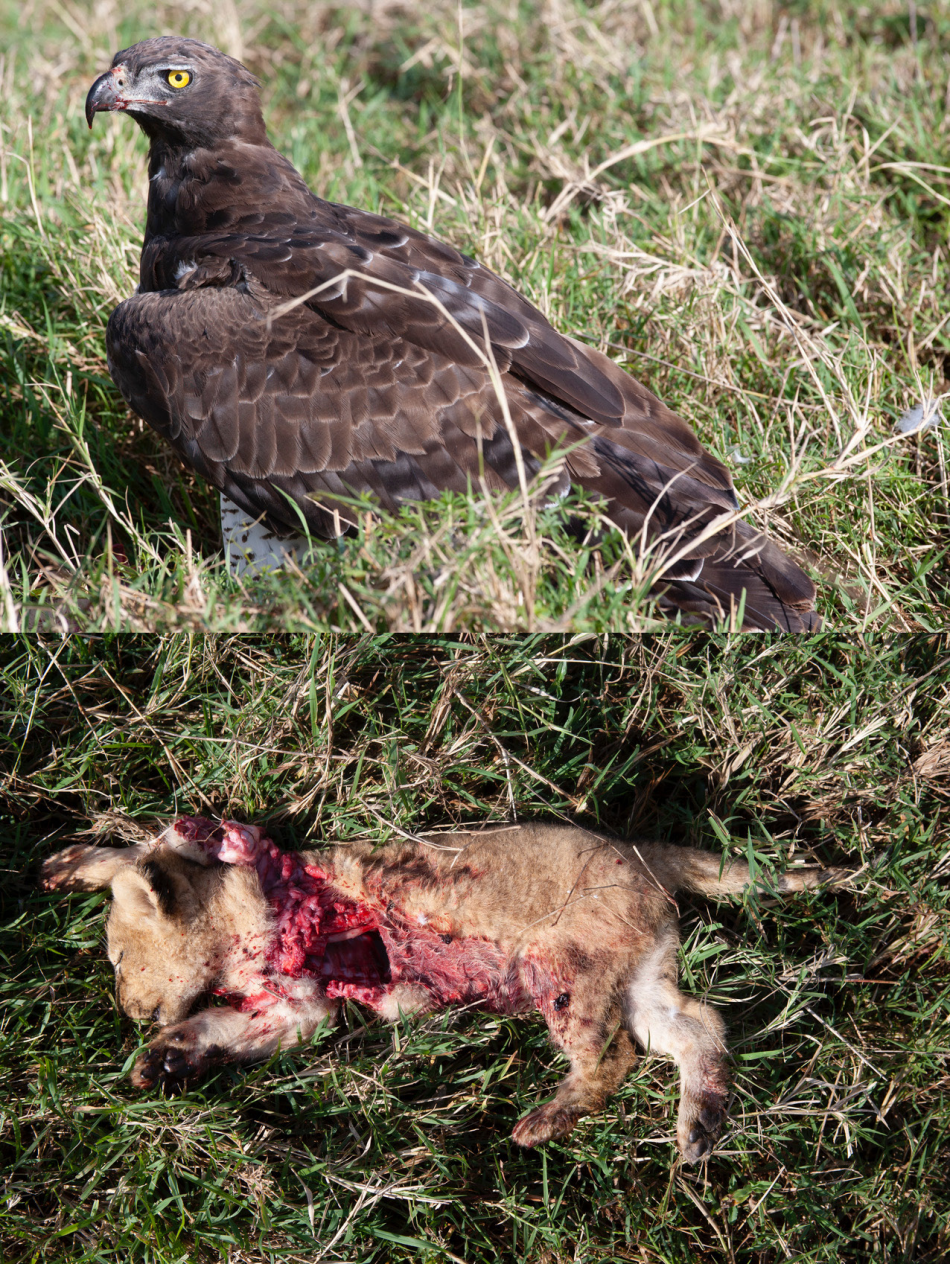
Top: An adult martial eagle perched with prey in long grass in the northern Mara Triangle, Kenya on 15 August 2008. Bottom: A close‐up photo of a ~6‐week‐old lion cub predated on by the martial eagle in the top photo on 15 August 2008. Note how the organs were fed on first and that the belly and back legs have been plucked. Photos courtesy of David Gulden.

**FIGURE 3:**
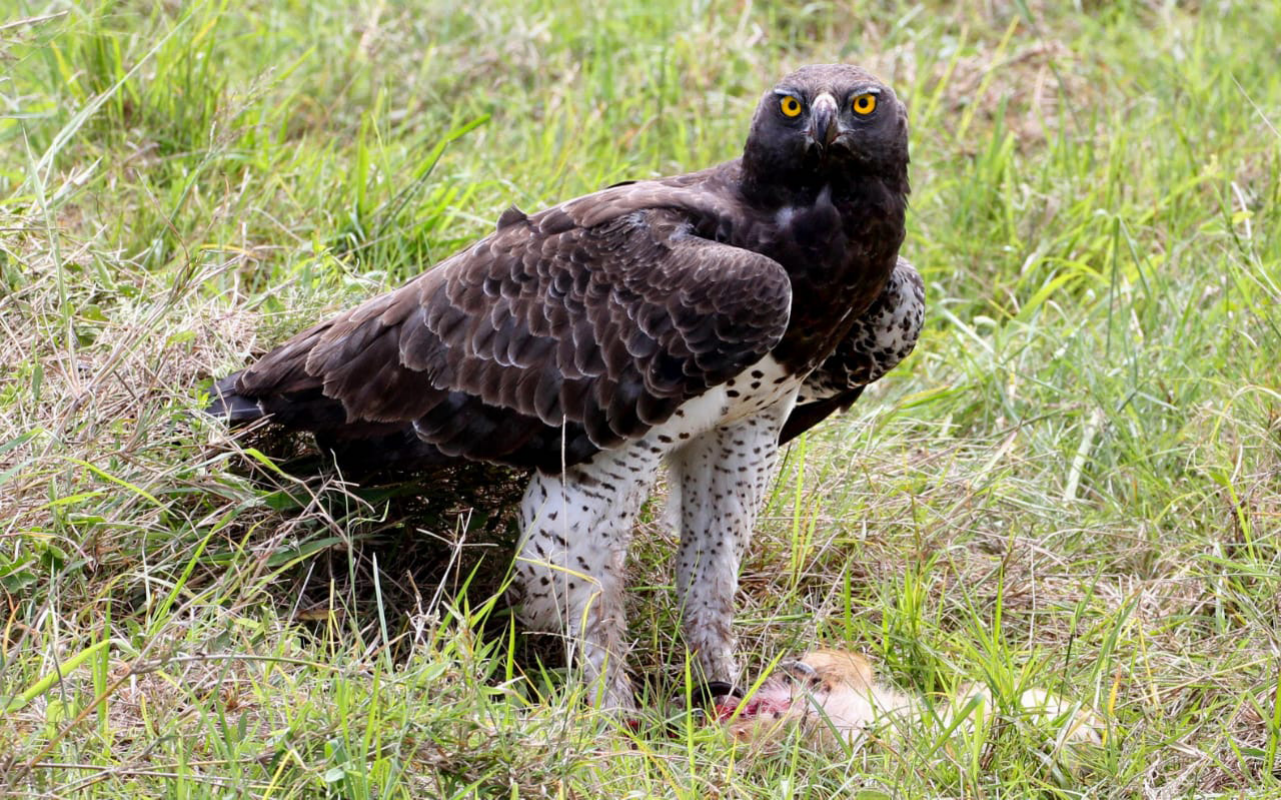
An adult martial eagle feeding on a young lion cub in the Maasai Mara National Reserve, Kenya on 14 February 2013. Photo courtesy of Patrick Reynolds.

**FIGURE 4:**
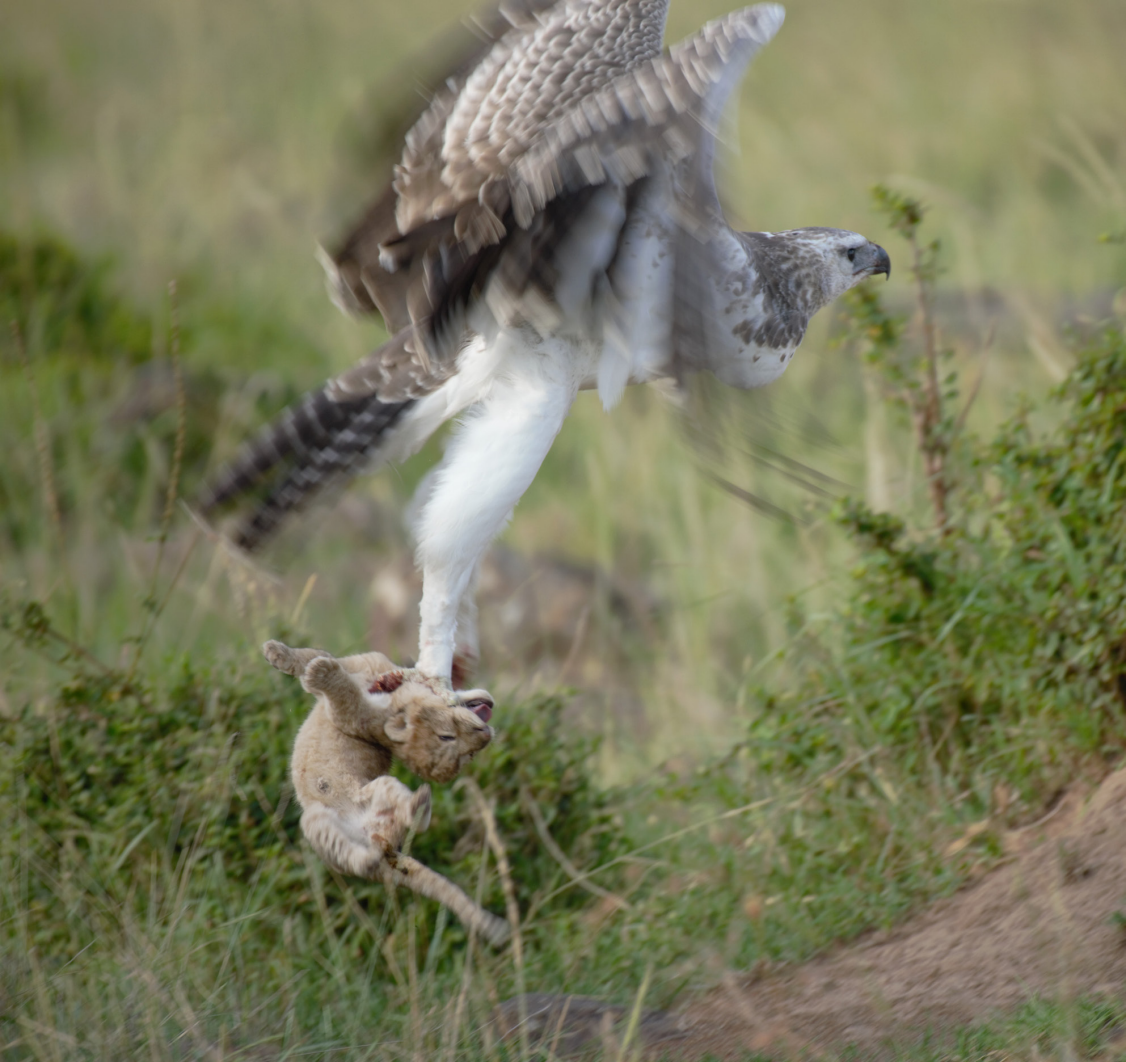
A recently fledged juvenile martial eagle taking off with a ~3‐week‐old lion cub on 15 March 2019 in Mara North Conservancy, Kenya. This juvenile martial eagle fledged from a known nest. The actual kill was not observed but based on the fledgling's behaviour we suspect that the adult female from this pair killed and provisioned this cub to the fledgling. Photo courtesy of Jes Lefcourt.

**FIGURE 5:**
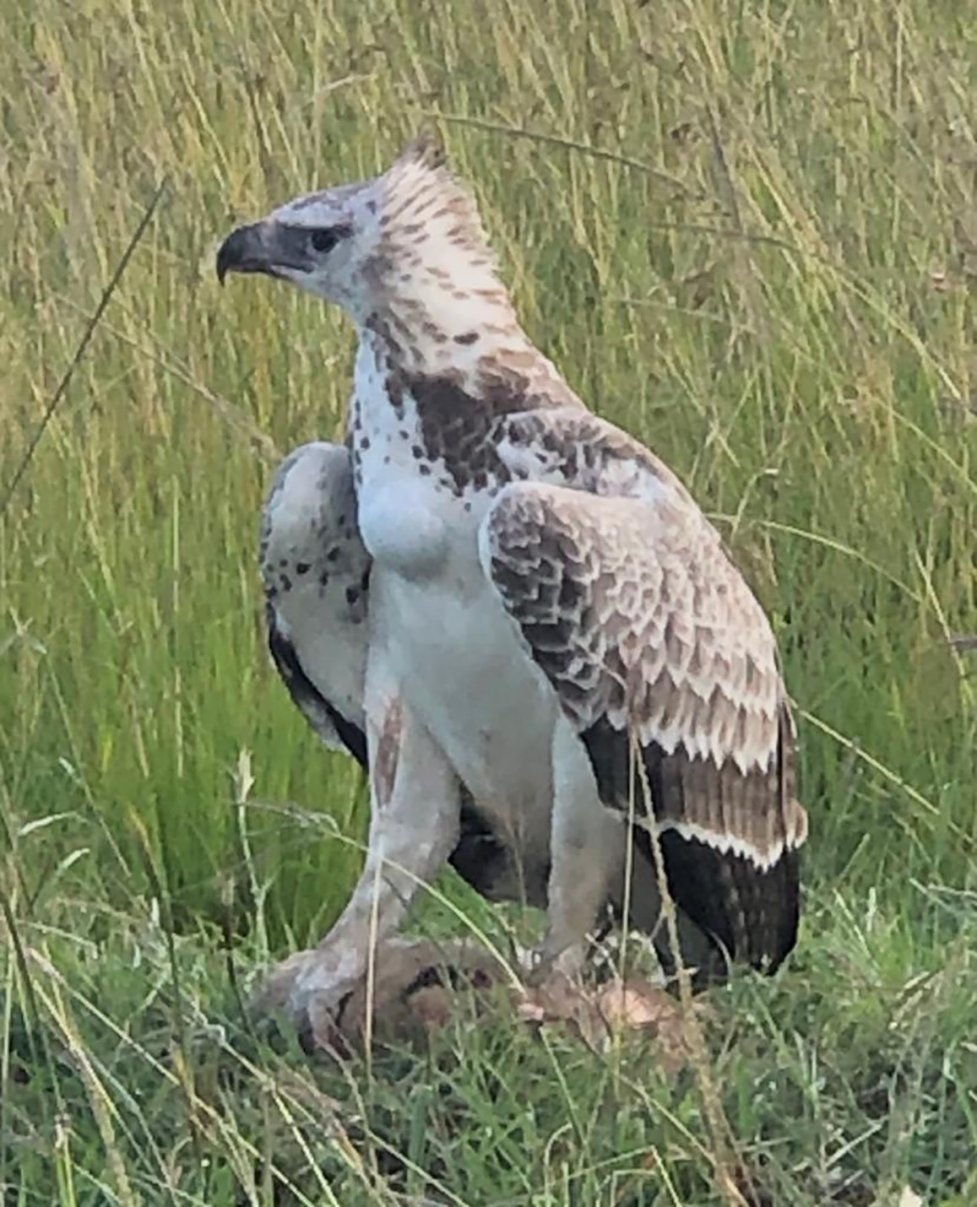
A juvenile martial eagle stands over a recently killed 4‐week‐old lion cub on 2 June 2023 in Lemek Conservancy, Kenya. Photo courtesy of Mike Saitoti.

1. On 15 August 2008, David Gulden photographed an adult martial eagle feeding on a ~6‐week‐old lion cub along a grass track in the northern Mara Triangle (Figure 2). As he approached the feeding eagle to photograph it, he inadvertently flushed the bird off its kill. This enabled close study and photographs of the freshly killed cub. Note how the organs were fed on first and that the belly and back legs have been plucked. This manner of feeding is typical for large eagles.
2. In December 2012, Patrick Reynolds and his guiding team from Governor's Camp reported an adult martial eagle persistently hunting and killing all three young lion cubs from a single cohort over a matter of weeks close to Musiara Marsh inside the Maasai Mara National Reserve. His field notes state that the martial eagle would follow the pride as they moved their cohort until all three cubs had been predated. It is important to note that while it is highly likely that all three kills are attributable to one territorial adult martial eagle, it cannot be ruled out that more than one adult eagle was involved.
3. On 14 February 2013, Patrick Reynolds again observed and photographed an adult martial eagle feeding on a young lion cub at lower Bila Shaka close to the Musiara Marsh inside the Maasai Mara National Reserve (Figure 3). As Patrick did not want to disturb the feeding eagle, we are unable to age the lion cub.
4. On 25 September 2016, Mark Mallone videoed a martial eagle attempt to take a ~6‐week‐old lion cub in Mara North Conservancy not far from Kicheche Mara Camp (Video 1). The attack was averted as the attending lioness leaped into the air, preventing the eagle from taking the cub.
5. On 15 March 2019, Jes Lefcourt photographed a recently fledged juvenile martial eagle holding a lion cub presumed to be ~3 weeks old (Figure 4). The observation took place in Mara North Conservancy not far from an area known locally as ‘Buibui’. The juvenile martial eagle is a known individual hatched from the nest of a well‐studied pair. Jes noted that the juvenile was calling repeatedly towards an adult female martial eagle perched nearby. This suggests that the cub was delivered to the dependent juvenile by the adult, rather than the juvenile having killed the cub.
6. On 23 September 2019, Patrick Reynolds and his guiding team from Governor's Camp observed an adult martial eagle hunting and killing a ~4‐week‐old lion cub in the Musiara Marsh inside the Maasai Mara National Reserve. The martial eagle could not fly well with the cub and was chased off its kill by attending adult lions that were resting nearby.
7. On 2 June 2023, Mike Saitoti from Fairmont Safari Club observed a juvenile martial eagle hunting and killing a ~4‐week‐old lion cub in Lemek Conservancy (Figure 5). Also a known individual, the martial eagle was approximately 9 months old (6 months since fledging) and had not yet dispersed from its natal territory. The cub was part of a cohort of four, attended by a lone adult male lion. The juvenile martial eagle struggled to fly with the cub and so dragged it to a termite mound to feed. Later in the day a lioness returned and moved the remaining three cubs to a different den. Over the following days, guides reported seeing the juvenile martial eagle watching the remaining cubs, but no further hunts were observed.

**VIDEO 1:** A lioness defends her ~6‐week‐old cubs from a martial eagle by leaping into the air attempting to swat the martial eagle on 25 September 2016 in Mara North Conservancy, Kenya. Video courtesy of Mark Mallone.

## 4 DISCUSSION

The records 4–7 were likely connected to at least four different martial eagle pairs. This is based on the detailed descriptions, videos and photographs provided by the observers and our knowledge of the territorial boundaries of telemetered martial eagles (Figure 1). This suggests that this behaviour is not simply learned or localised, but opportunistic and its frequency is likely linked to martial eagle and lion density as well as habitat.

Despite these records, martial eagle predation on lion cubs is almost certainly rare. An ongoing dietary study on martial eagles in the greater Mara region has amassed a dataset of over 1000 identifiable prey items. These records were collected using traditional systematic methods such as camera trap placements at nests during the breeding season and ground‐truthing kills identified by telemetry (Hatfield, 2018; Hatfield et al., 2024). This effort did not yield any records of predation on large mammalian predators, and all seven lion cub predation records presented in this manuscript were collated through anecdotal and opportunistic reports. To acquire additional records, one of the co‐authors (LP) received 20 responses from Mara‐based safari guides whom he had contacted to ask if they had ever witnessed this behaviour. None had.

Ongoing work in the Mara has shown that male and female martial eagles select for different prey items, with much larger females selecting for much larger prey (Hatfield et al., 2024). The estimated weight range of the predated lion cubs in the field records (2–6 kgs) (Smuts et al., 1980) strongly suggests that female martial eagles are predating on lion cubs. Unfortunately, sexing martial eagles using photos is challenging, but the images of the martial eagles included with the field records offer some support for this reasoning as they depict heavy‐set eagles, with thick tarsi, large feet and proportionally smaller eyes when compared to head size. Lion cubs are inherently a risky prey item for martial eagles as is evidenced by records 4 and 6. Larger female martial eagles can reduce this risk when compared to males, by being more capable of flying away with lion cubs from a kill site. That said, we think it is worth noting that the predation attempt videoed in record 4 is brazen and fraught with risk. We highly doubt that even the biggest female martial eagle could have killed and flown with the ~6‐week‐old cub in the video.

While lions are not important prey species for martial eagles, these records suggest that an individual martial eagle could have an impact on local lion population dynamics. The behaviour observed in December 2012, where a martial eagle appeared to repeatedly return to a cohort of lion cubs until all cubs had been eaten, is evidence of this. Whether these types of events have species‐level population impacts is highly unlikely, but in extreme circumstances where the personality and feeding preferences of an individual eagle lead it to repeatedly target cubs, an individual lion pride could be highly affected.

**FIGURE 6:**
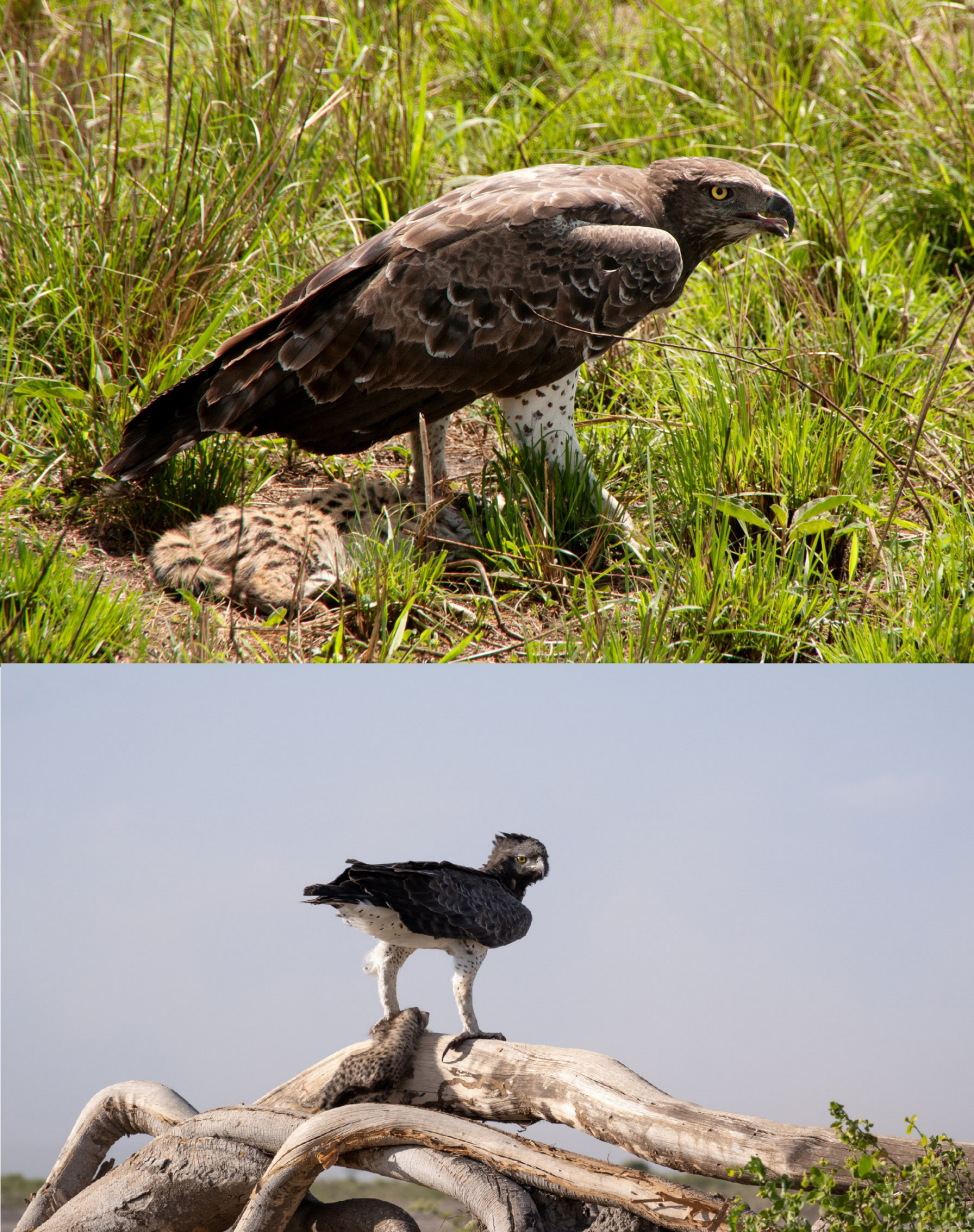
Top: A subadult martial eagle perched with a recently killed serval on 11 March 2013 in Queen Elizabeth National Park, Uganda. Photo courtesy of Bart Wursten. Bottom: An adult martial eagle perched with a ~4‐month‐old cheetah cub on 27 February 2023 in Amboseli National Park, Kenya. Photo courtesy of Andy Biggs.

**FIGURE 7:**
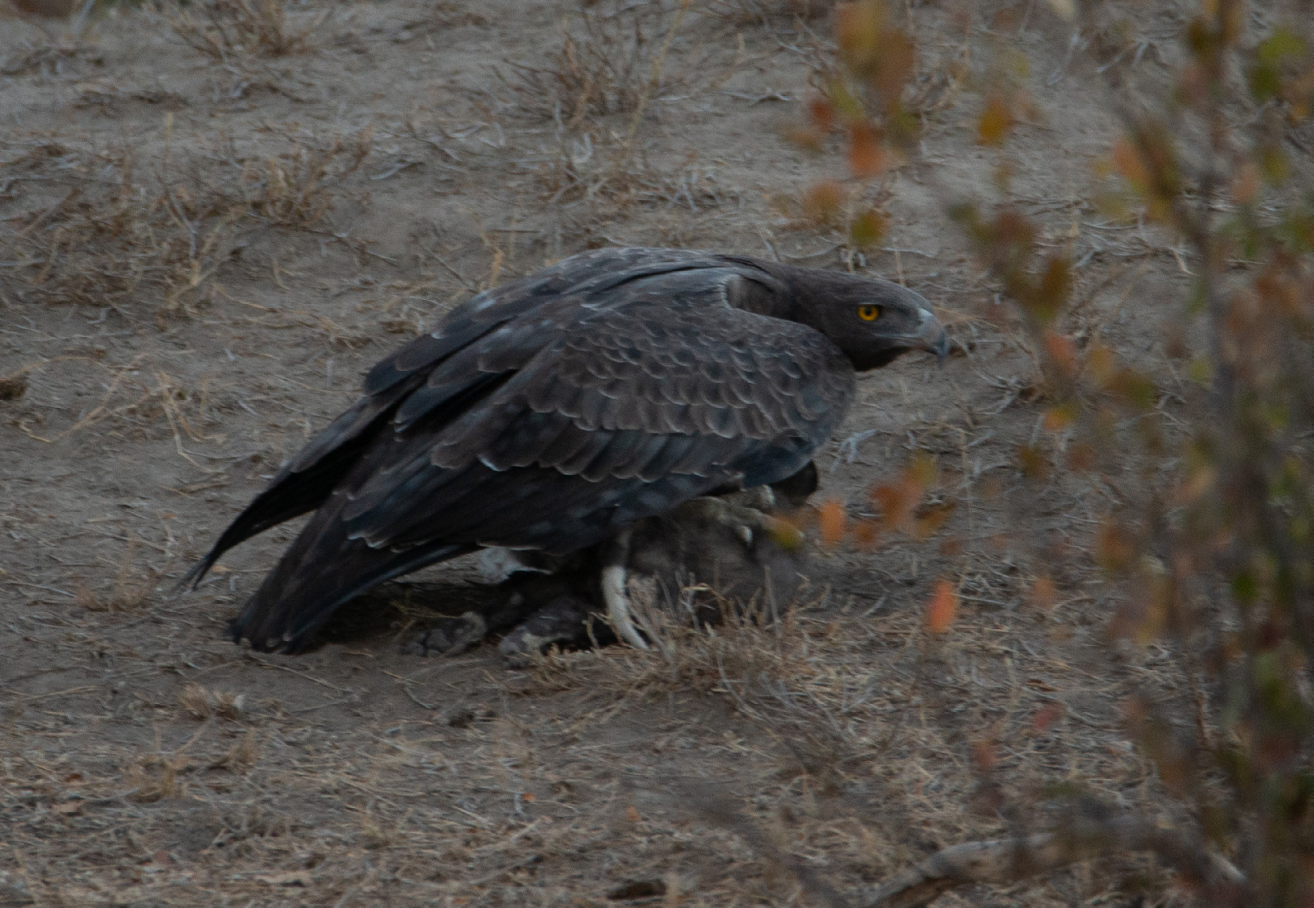
An adult martial eagle perched with a recently killed African wild dog pup on 21 July 2016 in Klaserie Private Nature Reserve, South Africa. Photo courtesy of Kevin MacLaughlin.

The seven records above are the first published detailed observations of lion cub predation or attempted predation by martial eagles, but they are not the first records of top avian predators killing large mammalian carnivores. A search of the literature yielded one brief mention of martial eagle predation of a lion cub, but no details were provided (Naude et al., 2019). There are also single records of martial eagles predating on a leopard (*Panthera pardus*) cub (Balme et al., 2013), caracal (*Caracal caracal*) kitten (Naude et al., 2019), a cheetah (*Acinonyx jubatus*) cub (Figure 6) and an African wild dog (*Lycaon pictus*) pup (Figure 7). There are several photographed records of martial eagles predating on both juvenile and adult serval (*Leptailurus serval*) (Figure 6). Golden Eagles (*Aquila chrysaetos*) have been recorded several times killing and eating brown and black bear (*Ursus arctos* and *americanus*) cubs (Sørensen et al., 2008) and wolf (*Canis lupus*) pups (Fernández‐Gil et al., 2024) and a Verreaux's Eagle Owl (*Bubo lacteus*) has been seen feeding on what was presumed to be a freshly killed lion cub (Taylor, 2017).

Given all this evidence, we suggest that this behaviour cannot be classed as interspecific killing or even intraguild predation (Lourenço et al., 2014; Polis et al., 1989). Instead, it likely represents pure predation where, in the case of our records, the martial eagle opportunistically views small lion cubs as a prey item and a source of food. These predation events are probably rare but widespread and underreported in the literature and therefore poorly understood. We therefore call upon observers to keep careful records and take photographs of any such predation events and to submit these to databases such as eBird and iNaturalist.

## AUTHOR CONTRIBUTIONS

**Richard Stratton Hatfield:** Conceptualization (equal); data curation (equal); visualization (lead); writing – original draft (equal); writing – review and editing (equal). **Lemein Parmuntoro:** Data curation (equal); writing – review and editing (equal). **Simon Thomsett:** Conceptualization (equal); writing – review and editing (equal). **Patrick Reynolds:** Conceptualization (equal); writing – review and editing (equal). **Nicholas B. Elliot:** Conceptualization (equal); writing – original draft (equal); writing – review and editing (equal).

## CONFLICT OF INTEREST STATEMENT

The authors have no competing interests to declare.

## Data Availability

All data presented in this manuscript are included in the manuscript.
